# Supplementary figures and images for: Imputed gene expression risk scores: a functionally informed component of polygenic risk
Source: Hum Mol Genet. 2021 Feb 22;30(8):727–38. doi: 10.1093/hmg/ddab053 (PMC8127405; doi:10.1093/hmg/ddab053)

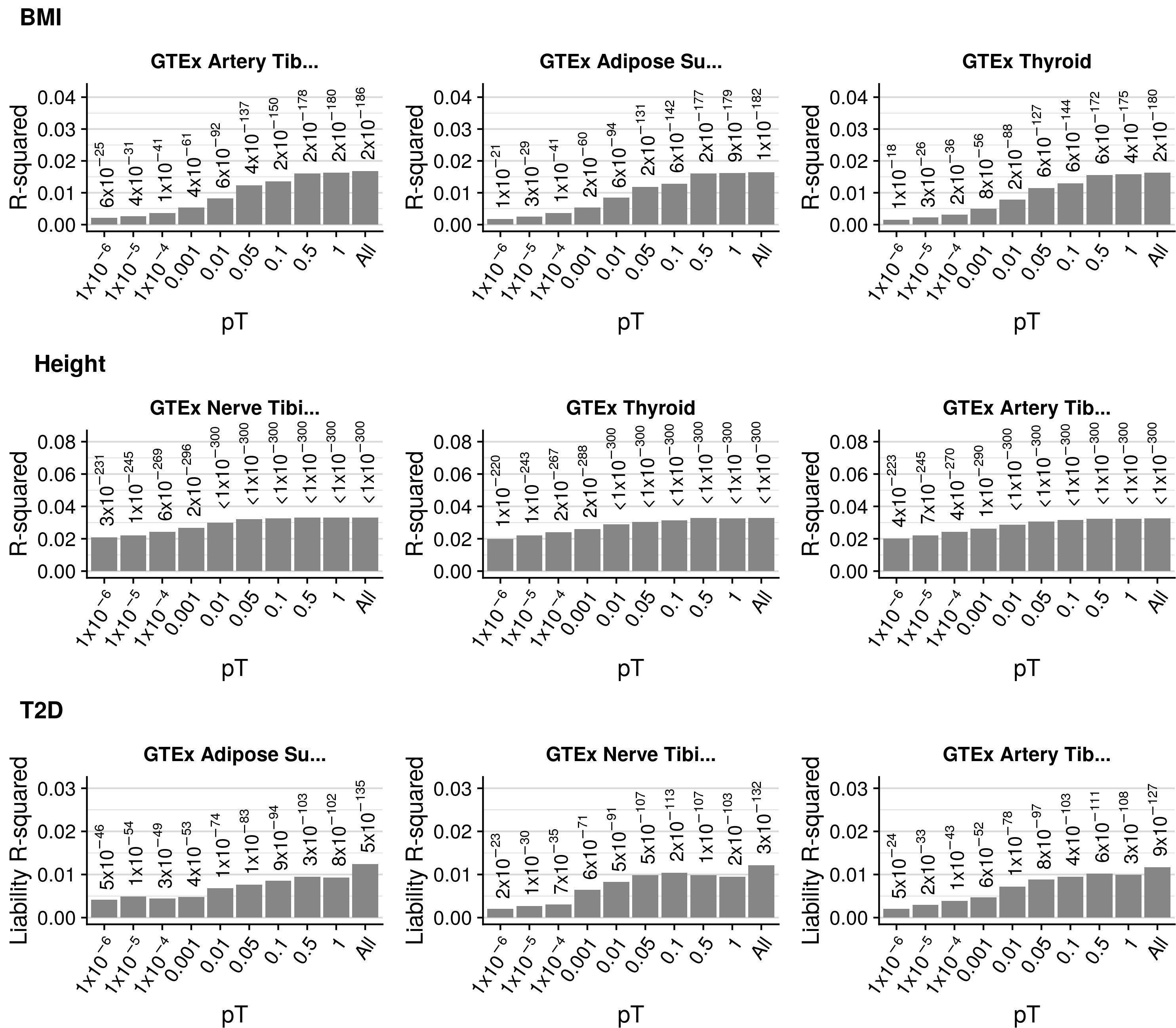

Supplement: GeRS_HMG_R1_Fig1_bw_ddab053 [file gers_hmg_r1_fig1_bw_ddab053.jpeg]

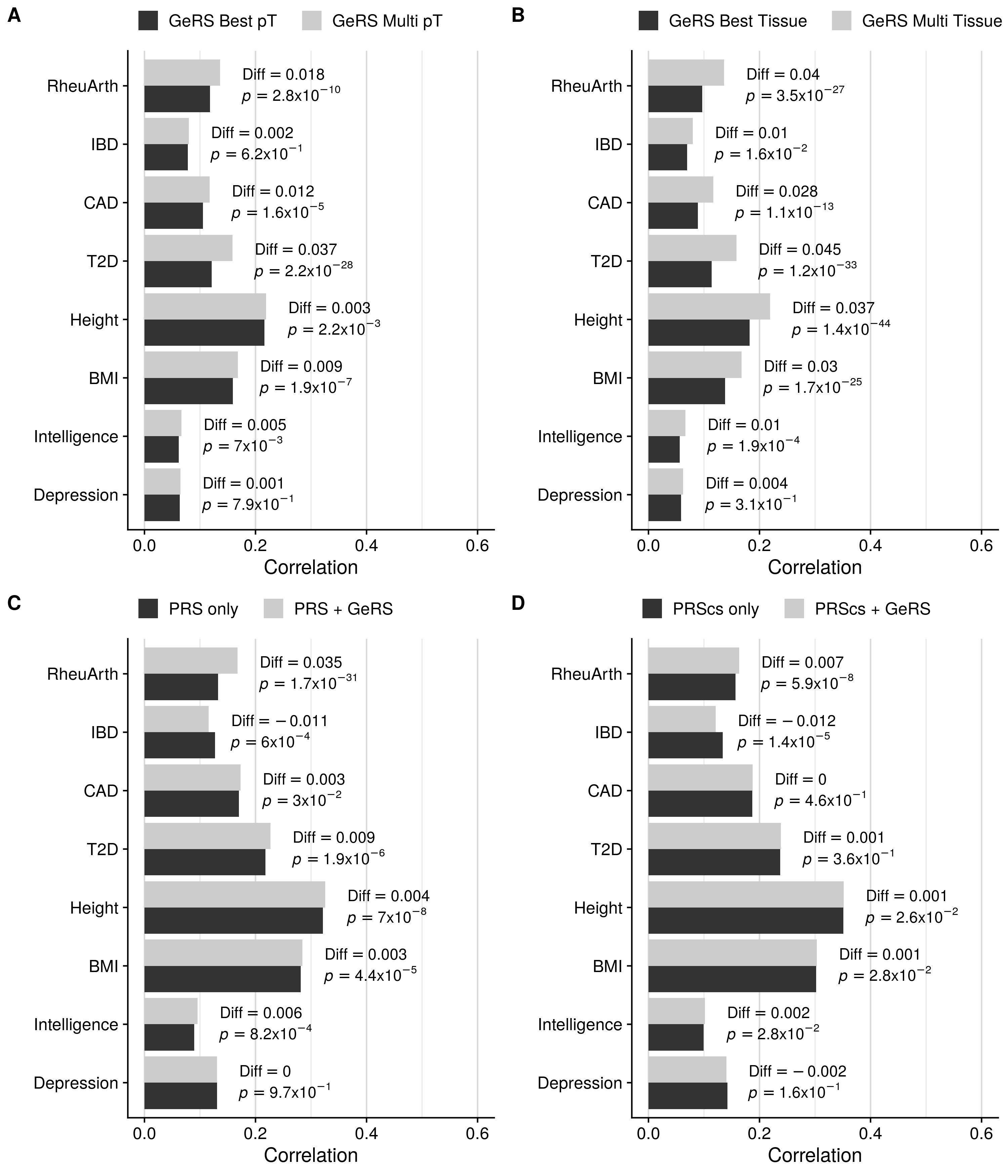

Supplement: GeRS_HMG_R1_Fig2_bw_ddab053 [file gers_hmg_r1_fig2_bw_ddab053.jpeg]

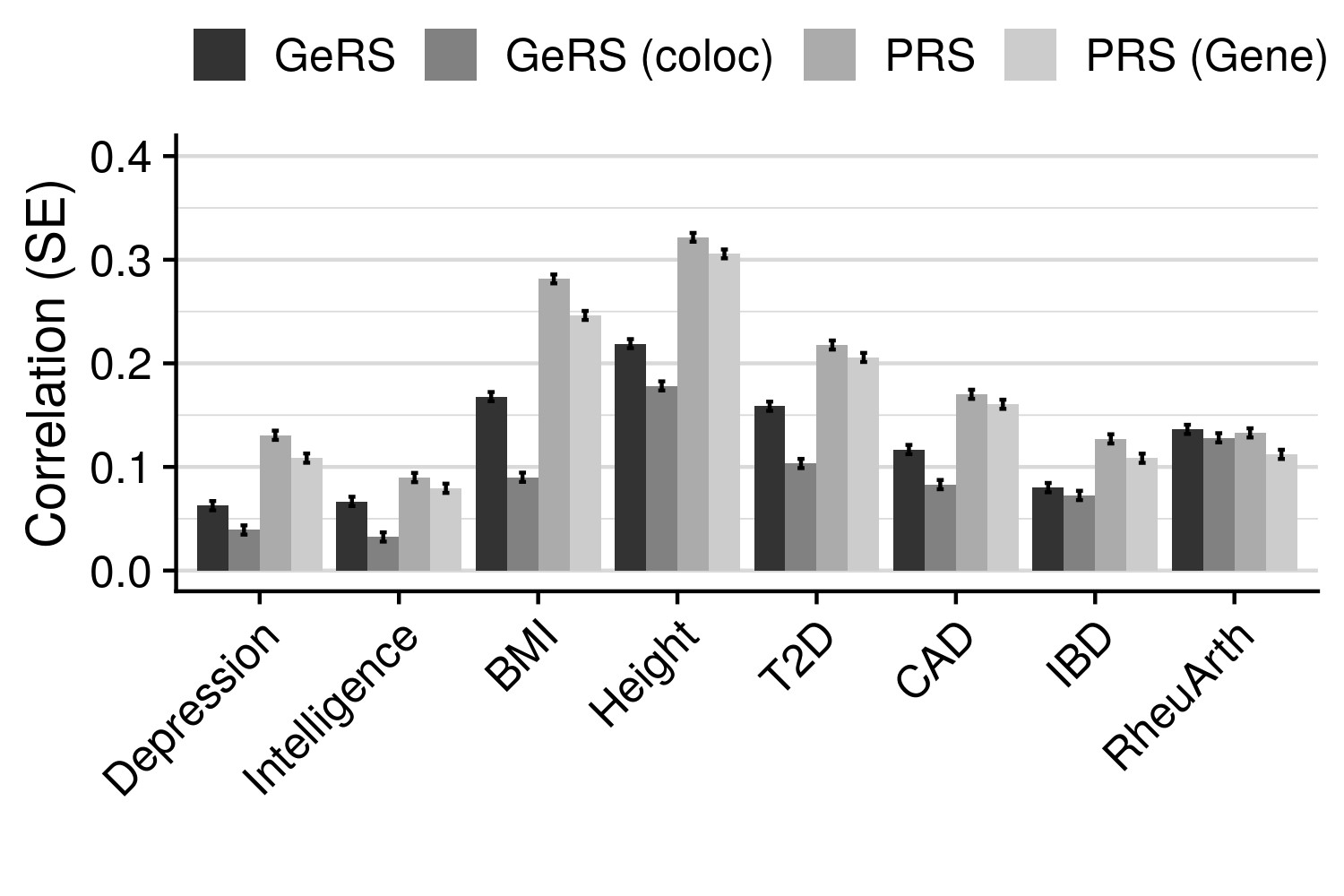

Supplement: GeRS_HMG_R1_Fig3_bw_ddab053 [file gers_hmg_r1_fig3_bw_ddab053.jpeg]

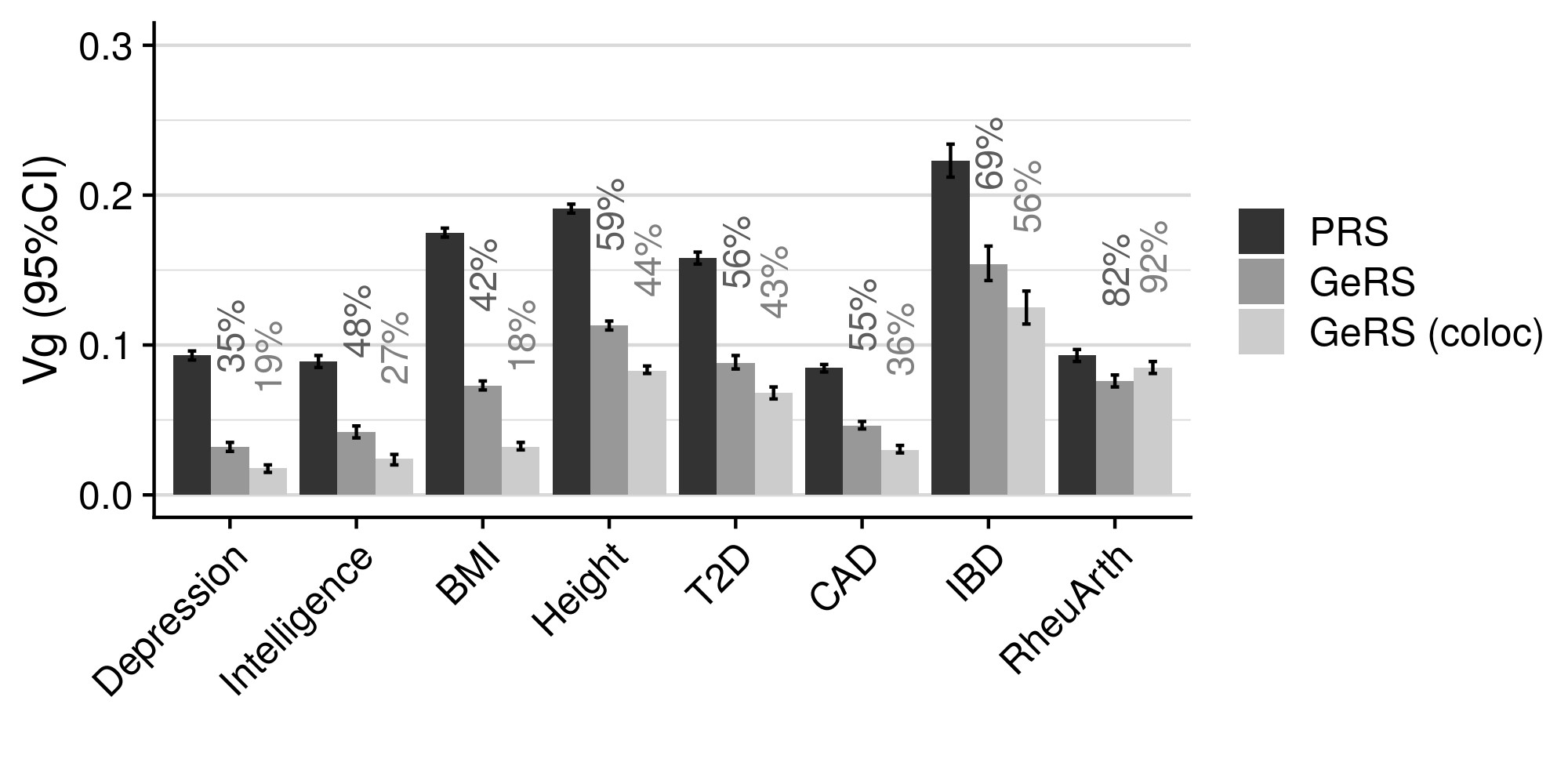

Supplement: GeRS_HMG_R1_Fig4_bw_ddab053 [file gers_hmg_r1_fig4_bw_ddab053.jpeg]

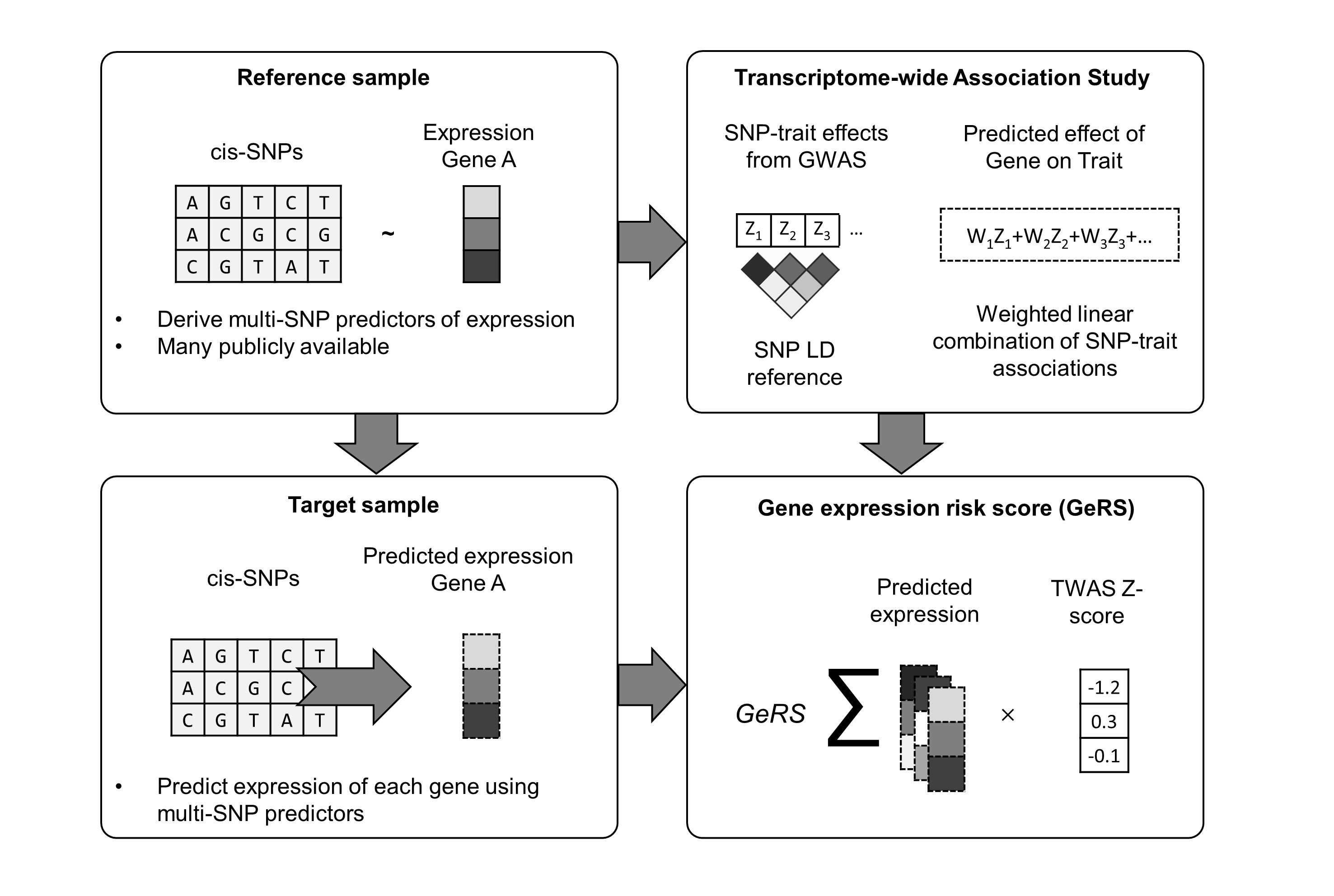

Supplement: GeRS_HMG_R1_Fig5_bw_ddab053 [file gers_hmg_r1_fig5_bw_ddab053.jpeg]

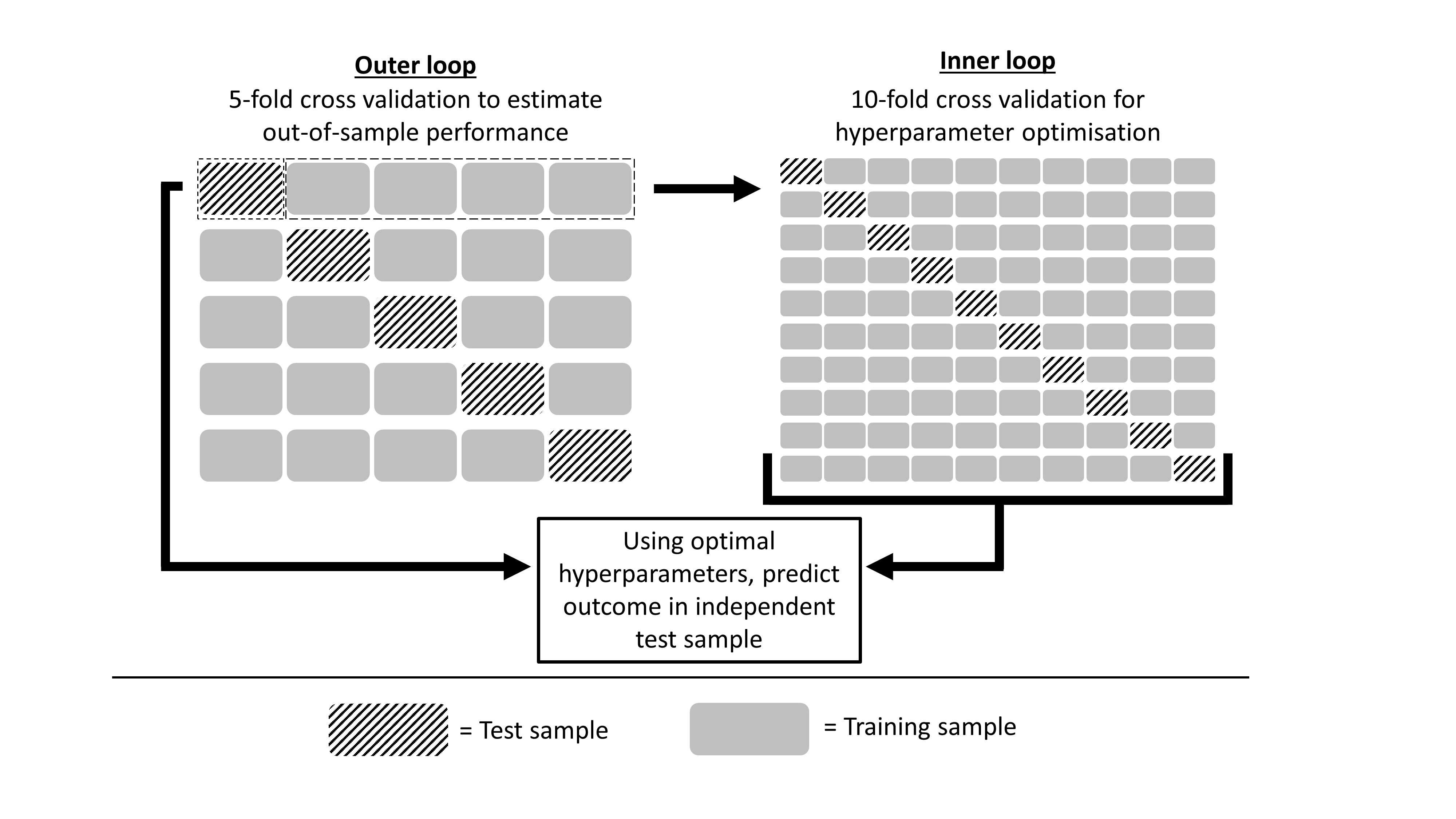

Supplement: GeRS_HMG_R1_Fig6_bw_ddab053 [file gers_hmg_r1_fig6_bw_ddab053.jpeg]
